# Editorial: Psychosis and Personality Disorders: Do We Need Early Diagnosis for Successful Treatment?

**DOI:** 10.3389/fpsyt.2020.00584

**Published:** 2020-06-16

**Authors:** Silvio Bellino, Paola Rocca

**Affiliations:** Department of Neuroscience, School of Medicine, University of Turin, Turin, Italy

**Keywords:** psychotic disorders, personality disorders, early onset, predictive factors, outcome

Early diagnosis is one of the most relevant issues of modern clinical psychiatry. Several investigations pointed out the need to detect the prodromal signs and symptoms of psychiatric diseases to define specific monitoring and early interventions strategies. In particular, the lack of diagnosis and treatment in the early periods of disorders that present their onset in adolescence or young adulthood, such as schizophrenia spectrum disorders (SSDs) and severe personality disorders (PDs), deals with a high level of disability and a worse illness course.

Many patients who suffer from psychosis at the first contact with medical care present multiple risk factors to develop psychosis, such as positive family history for psychosis, pregnancy, or birth complications, early traumatic events, substance use, and mental disorders predisposing to the onset of psychosis. A careful search for detection of these factors would be important to prevent significant negative consequences in terms of psychosocial functioning.

In a similar way, although severe PDs, in particular borderline personality disorder (BPD), are known to have their onset in young age, their diagnosis and treatment are usually delayed. It is of fundamental importance to identify clinical conditions that can evolve in BPD, such as disruptive behaviour and disturbances in attention and emotional regulation, conduct disorders, oppositional defiant disorder, attention deficit-hyperactivity disorder, and substance use.

In the past decades, there was a lively debate to establish whether pharmacotherapy in the prodromal phases of psychiatric disorders is efficacious and ethically acceptable, but final conclusions have not been drawn. On the contrary, it is commonly accepted that specific psychosocial interventions that involve patients' family members produce positive results in terms of reduction of symptoms, comprehension of disorders and improvement of coping skills. These results can be particularly useful if we consider that, already during the first phases of monitoring and treatment, the risk of service disengagement and medication non-adherence is high and should be carefully faced.

The Editorial is aimed to make clear what are the main topics addressed by the articles of this Special Issue on early onset of psychosis and personality disorders. Many authors focused their contributions on identification of young individuals at high clinical risk for psychosis (CHR-P). In particular, Montemagni et al. reviewed studies that proposed or examined a model of transition to psychosis of subjects with high clinical risk. Authors found that only few studies performed an internal validation of models and only biological and neurocognitive models received validation. So, the validation process of predictive models is still at the initial stage. To promote further research on CHR-P state in children and adolescents, Molteni et al. proposed a longitudinal protocol study with the aim to measure transition to psychosis or other psychiatric disorders after 2 years and to investigate the predictive value of specific clinical, neuropsychological, and neuroimaging factors on prognosis. A core issue of studies of young individuals at risk for psychosis is to improve the ability to detect these subjects at an early phase, before the onset of a first psychotic episode. In order to obtain this goal, Fusar-Poli et al. developed a clinically based, transdiagnostic risk calculator and performed a study to support the implementation of this tool in the real-world clinical practice.

A relevant question concerning early detection and treatment of schizophrenia spectrum disorders (SSP) is why successfully treated patients still present social functioning impairment. Armando et al. suggested that functional impairment derives from arrested development of social cognition during adolescence and early adulthood, particularly of reflective thinking processes defined as mentalization.

Other contributions included in this Special Issue addressed questions concerning prediction and early detection of personality disorder. Bozzatello et al. examined literature studies in order to identify factors (precocious environmental factors, temperament and personality traits, early psychopathological features, and neuroimaging factors) that are related to high risk of early onset borderline personality disorder. Boldrini et al. investigated the relationship between personality disorders and CHR state. Authors found that personality disorders were present in almost 40% of CHR patients and the most common were schizotypal and borderline personality disorder. However, the studies investigating the effects of baseline personality diagnoses on transition to psychotic disorders obtained insufficient and contradictory results. Other two contributions examined the relationship of personality disorder with psychosis. In particular, Cavelti et al. compared cognitive, emotional, and behavioral responses to verbal hallucinations in youth with BPD versus schizophrenia spectrum disorders (SZ). Results replicated in BPD young patients the link between negative appraisal of voices and depression that has already been indicated in patients with SZ. Schultze-Lutter et al. considered the historical and phenomenological link of schizophrenia spectrum personality disorders, in particular schizotypal personality disorder (SPD) and psychotic disorders. This link was reassessed on the basis of recent evidence and authors concluded that SPD and psychotic disorders are not simply states of different severity on one common but on qualitatively different dimensions. The negative dimension would be predictive of SPD, the positive of psychosis. So, the assessment of multiple schizotypy dimensions would be an essential step for early differential diagnosis.

Other authors considered issues related to pharmacotherapy and psychosocial interventions for schizophrenia. Ringen et al. investigated clinical predictors of antipsychotic dose reduction or discontinuation in the first year of treatment in schizophrenia versus bipolar disorder. As treatment guidelines recommend to avoid dose reduction or discontinuation of antipsychotics in the first year, identification and differentiation of predictors between affective and non-affective psychoses is of central importance for clinical practice. Authors found a dose reduction in the first year in both first treatment groups across diagnoses, but predictors were different in the two groups (weight increase was a predictor in schizophrenia, baseline severity of symptoms predicted dose reduction in bipolar disorder). Deste et al. considered in their contribution the effects of a psychosocial intervention, cognitive remediation, in patients with schizophrenia, in order to verify whether cognitive deficits are more sensitive to remediation in early than in chronic schizophrenia. Results indicated a greater improvement of clinical and functional measures in early course patients compared with chronic patients, while no difference between groups was found in the neurocognitive parameters.

Another relevant issue concerning outcome of patients with first-episode psychosis was addressed in the article proposed by Weibell et al. These authors evaluated the long-term association between substance use and cognitive functioning in a large sample of first-episode psychotic patients. What is the effect of early substance use cessation on cognitive trajectories of these subjects? Patients who stopped using substances in the first 2 years improved on some cognitive measures, especially motor speed and verbal learning indices, while control groups did not.

In summary, this Special Issue presents a series of valuable contributions that deal with recent evidence on risk factors, early detection, and clinical and functional outcome of young patients with psychosis and personality disorders. In some cases, data are promising and can help the clinicians to improve their ability to detect subjects with high clinical risk and to obtain an early diagnosis with positive effects on outcome. More often, available evidence is insufficient, and further studies are required. So, this field of psychiatric research is certainly one that deserves significant efforts to confirm initial findings and can produce new knowledge with relevant implications for clinical practice.

## Author Contributions

All authors contributed to prepare the Editorial.

## Conflict of Interest

The authors declare that the research was conducted in the absence of any commercial or financial relationships that could be construed as a potential conflict of interest.

